# Devergie on Baths, Etc.

**Published:** 1855-02

**Authors:** 


ART. III. — *M. Devergie on Baths, etc.* Translated from the French, by Prof. Flint.

(Concluded.)

Physicians in general are but little acquainted with the potency of vapor baths and douches. Originated at the St. Louis Hospital, instituted under the direction of Darcet, these baths have extended thence into Paris until there exists perhaps twenty establishments in the city. One can estimate the efficaciousness of these baths by what has been done at the St. Louis Hospital, and what is now done there. Formerly numerous equipages were to be seen standing at the hospital gates having brought there some of the wealthiest of our citizens who came to seek in these baths relief of their sufferings. Now of more than 150,000 baths prescribed for our patients, 100,000, perhaps, are vapor baths. To the above statement it is to be added, they are taken daily by more or less of the 800 inmates of the hospital. If to this enormous number are added the vapor baths administered at the *Maison de Santé*, and the hospital *de la Charité*, and at the hospital *Beaujon*, as well to patients in the hospital as to out-patients, and then those which are taken at public establishments in the city, *Tivo'i*, *Les Néothermes*,

*Baines Sanite-Anne*, *Bains d'Alger*, etc., etc., the sum total will bear a large ratio to the population.

These baths, at the present time, are preferred by many persons to baths of water. One experiences, in fact, from a Russian bath a greater degree of comfort than can be procured from a simple bath, especially in winter, when it promotes the functions of the skin, gives suppleness to the limbs and relaxes the tissues stiffened and contracted by the cold. But this revolution which has taken place as respects the choice of baths is confined to the city; it has not extended to the country, and this is so far the fault of physicians, that two of the chief commercial cities in France may be cited, in which vapor baths having been instituted, the patronage is so little that a bath does not cost less than six francs, while in some establishments in Paris it has been reduced to sixty *centimes* (about twelve cents). I may be permitted, then, to enter into some details which will lead to a comprehension of their efficacy and importance. I shall be happy if I am able to induce my brethren in the departments to recommend the employment of these baths and to favor their establishment, since thereby I shall have rendered an immense service to the people.

And, in the first place, what is a vapor bath? What are the several varieties of vapor baths, portable, sweating, Russian and Turkish? A vapor bath has for its object to produce a copious artificial perspiration. This sweating, when it is produced by dry heat, constitutes a fumigation (*en boite*) which may be aromatic, sulphurous, mercurial or simple. These fumigations may be made in cubic boxes, called *à la Darcet,* in which the patient is seated, having the head only without the box, protruded through a hole made for that purpose in order that the respiration may be free. Under these circumstances the perspiration is produced by the stimulant effect on the skin of the caloric of the atmosphere in which the body is placed. In certain establishments for the use of mineral waters, an analogous effect is obtained by means of water which escapes from the earth, having a temperature raised to 50, 60, and 70 degrees (*centigrade*). (120°, 140°, 158° Fahr.) Thus at *Néris* at *Aix en Savoive,* and in other localities there have been constructed over the springs, which are surrounded by walls like those of wells, a series of *cabinets* into which air warmed by the water enters, producing rapidly copious perspiration. To a sweating bath of this description is generally applied the title of a *hell.* The conditions here are very superior, and with a temperature considerably lower perspiration is produced more rapidly, more abundant, and with less discomfort than in the boxes. However the details upon which we are about to enter will prepare us to understand the superiority of vapor baths over the preceding, and over those called portable. In the fumigating boxes sweating is produced entirely by the action of heat on the skin, and the higher the temperature is raised, the more the patient transpires. But this artificial heat diffused over the surface affects powerfully the respiration, occasions a tendency of blood to the lungs and head, bringing on palpitations and cephalalgia. When, on the other hand, the patient respires the heated air which is to produce sweating, the pulmonary exhalation favors singularly the transpiration from the cutaneous surface, and the circulation is less excited. So true is this, that it suffices to place the face over a vase containing hot water in order for the whole body to be bathed in perspiration.

Since, then, pulmonary exhalation induces speedy sweating on the surface, and without any inconvenience, it follows that a conjoined action upon the skin and the pulmonary mucous membrane, must be much more conducive both to the production of sweating, and the comfort of the patient.

As there are portable vapor baths, of which I shall frequently speak, so there are small apparatus for generating hot air, designed as a substitute, in a measure, for the fumigating boxes. The council of public health and salubrity of the Seine, at the epoch of the cholera in 1849, entertained the project of furnishing to the poorer classes a means potent, rapid in operation, and cheap, for inducing reäction in the algid stage of cholera. M. Cadet de Gassicourt proposed an apparatus very simple, which was adopted, and which is still employed in most of the hospitals of Paris, and especially at the St. Louis Hospital where it is often an object to produce copious sweating in cases of repercussion of cutaneous diseases, in rheumatic affections, as well as in choleraic accidents. This apparatus consists of a cone of sheet-iron somewhat larger than a sugar loaf of the largest size, having at the lower part a small door through which is introduced a spirit-lamp with three wicks, the cone terminating at the summit in a tube of sheet-iron, with an elbow, enlarged and funnel-shaped at the extremity. The tube admits of being elongated and shortened at will, the ends of the portions of which it is composed entering into each other. To use this apparatus, designed for patients who cannot leave the bed, the cone is placed on the floor, the pipe with the elbow attached to it, and the extremity of the latter, introduced into the bed. The bed-clothes are kept raised from the body by means of half hoops, such as are used in fractures, placed crosswise and along the length of the bed. Matters being thus arranged, a spirit-lamp is placed within the cone, the three wicks are lighted, and when the patient is in full perspiration, if the heat be too great, one or two of the wicks are extinguished by means of small covers made for that purpose. In six or seven minutes perspiration is usually established, and it is practicable to regulate its duration, and to graduate it as may be desired. It facilitates the end to cause the patient to drink water at a temperature neither warm nor cold.

Coming to the subject of vapor baths, these are taken in a sweating-room (*bains en étuve*) or they are portable. The latter have been extensively used, having the advantage of being taken by the patient in bed, or in a chair in his own chamber. Moreover, a small apparatus costs but a small sum. The apparatus, *Adam*, consists of a sphere of copper terminating with a tube of 25 to 30 *centimètres*. Water in the sphere is heated by a spirit-lamp, and aromatic plants are placed in the water.

Another apparatus is on the same system, but with modifications in form. It consists of a sheet-iron vessel holding from two to four pints, which is placed on a charcoal furnace, and ebullation produced under a pressure somewhat greater than that of the atmosphere, so as to furnish, by means of a long tube, vapor at will, and at any given point in an apartment. A pipe connected with the heating apparatus is introduced into the chimney. This apparatus distributes as much vapor as may be desired, while others either fall short of the requisite quantity, or heat the patient too much. It is much more expensive, costing from eighty to one hundred francs, while the cost of the others ranges from twenty to fifty.

All these contrivances for vapor baths are mere playthings. They all produce perspiration by elevating the temperature of the skin, and consequently the sweating is difficult, more or less fatiguing, and in no degree comparable to a bath in a sweating-room.

The bath in a sweating-room (*d'étuve*) requires a collection of apparatus suited not to give one bath only, but a certain number daily. And, first, is necessary a generator of vapor, or a steam boiler, in which water may be raised to 120 degrees (*centigrade;* 270° Fahr.) of temperature. From this *generator* issues a tube leading to a small chamber ordinarily constructed of wood, of a cubic space from 10 to 12 *mètres*, (a little over 32 feet). In this chamber is a bed of wood, or cane, on which the patient lies. Beneath this camp-bed, toward the foot, is situated the stop cock regulating the escape of the vapor. The latter should never be left to the control of the person taking the bath, but should be in the hands of an attendant who does not leave the patient. A centigrade thermometer is placed so as to be seen by the patient, and it should be of alcohol colored red, upon a white ground, in order that the degrees which it denotes may be very conspicuous. Within reach of the patient, also, should be placed a basin in which water will not remain, running off through a waste pipe. This basin should contain a large sponge, and situated above it a tube conducting cold water regulated by a stop-cock. The sponge thus imbibing water constantly renewed, is designed to be used in moistening frequently the forehead and face of the person taking the bath to obviate congestion of the head.

In a part of the room opposite to the camp bedstead, toward the ceiling, there should be a large reservoir for showering, capable of furnishing a shower of rain of some duration. Into this reservoir should enter a tube for warm, and one for cold water, which admit of being opened separately by means of bent levers with wooden handles falling within reach of the person taking the bath, or the attendant, by means of which may be given at will a *douche* of warm, or of cold water, or of warm and cold water commingled. It should be a shower of water rather than a *douche*, and it answers very well to have two reservoirs a little elevated, the one filled with warm, and the other with cold water.

The door of the sweating-room should open both ways, and be without latch, or any fastening, so that in case of accident the person taking the bath may be able to get out by pushing the door before him. This door, moreover, should have inserted in it a large pane of glass permitting the attendant to see what is going on within the room in case he should be momentarily without. Such a collection of apparatus has tended to place it beyond the thoughts of many to institute baths of this kind on account of the first cost. The expense, however, is little or nothing when the mode of production is well understood, and when the steam is applied to other purposes. A very small boiler suffices to heat a large mass of water, and a little fire only is needed to heat a small generator of steam. Hence, economy is consulted in heating all the water used in a bathing establishment by means of steam thus developed, so that the *generator* shall not be exclusively for the vapor baths at the same time that they may be given at any moment without the necessity for the expenditure of heat specially for each bath.

Finally, near the sweating-room there should be a *cabinet* containing a couch on which the patient may repose, provided with coverings sufficient to develop and maintain sweating. Hospitals, in place of a room suited to contain but a single patient, should be provided with a larger apartment, in which are arranged semicircular steps on which the patients seat themselves; and in proportion as the patient takes a higher position in this sort of amphitheatre, the temperature is more elevated, the vapor, in proportion to its heat, rising to the upper part of the room.

Resting here, I will proceed to indicate how vapor baths are to be taken.

With respect to this point varieties are distinguished by the names of *simple baths; Russian baths; baths with shampooing, anointing, etc., called Turkish baths.*

*Different modes of administering vapor baths.*—The most simple bath consists in placing the patient, nude, on a camp bed, the sweating-room, by the previous emission of vapor, being heated to 35 degrees centigrade, (95° Fahr.) After some minutes the temperature is raised, gradually, by opening the stop-cock to admit the vapor one half, so as to increase, successively, to 38, 40, and 42 degrees, (100°, 104°, and 107° Fahr.) This temperature is sufficient for persons of a lymphatic or lymphatico sanguine temperament who sweat readily. Once produced, it may be maintained by opening, from time to time, the stop-cock, or by leaving it open a sixth or an eighth of its diameter. This is the temperature suited for all patients affected with moist or secreting affections of the skin. By raising the temperature above this point, the skin is irritated, and the condition of the affected parts is aggravated, instead of being relieved.

A person sweating with more difficulty requires a higher degree of temperature; but, under these circumstances, I prefer not to produce perspiration until the third or fourth bath, and to maintain the low temperature. When, however, it is employed in rheumatic affections, in which it is not only necessary to obtain sweating, but is useful to produce a certain excitation of the skin, the temperature of the room may be carried to 50 or 55 degrees, (122°, 131° Fahr.) The latter is customary at all the vapor baths in Paris. It is very injurious for diseases of the skin, and I have often much trouble in obtaining a decrease of temperature for my patients. Moreover, a vapor, as well as a water bath, should be agreeable to the patient, and whenever it induces notable palpitations, headache, or a sensation of heating in the head, it is too hot.

The duration of the bath is usually from twenty to twenty-five minutes.

The bath taken as just mentioned is less favorable than when modified as follows: After ten minutes, the patient is placed under a shower of water, at first of moderate temperature, and cooled by degrees, the whole time occupied being from one to two minutes, the patient then being replaced on the camp bed. The same *douche* is again given after ten minutes; and, finally, a *douche* of very warm water is applied to the feet.

After the bath the patient is clothed in a heated dressing-gown and a woollen cloak; the head and ears being enveloped in a hot towel. He is placed on the couch, in an extended position, the arms lying alongside the body, the feet and limbs encased in a very warm towel, the body rolled in several coverings, and left to himself. Free sweating ensues, and after twenty minutes he should be directed to separate his lower limbs, and remove the arms a little from the body, in order to diminish the perspiration. He remains three-quarters of an hour on the bed, when he dresses himself, leaves the establishment, and takes a brisk walk to keep up a moisture on the surface, or if this course be not pursued, it is best that he be taken home in a carriage.

In the Russian bath the patient is heated rapidly by applying vapor raised to 55 degrees of temperature, (131° Fahr.); and after some minutes he is placed under a *cold douche*. The sweating and the *douche* are repeated four or five times in the space of twenty or twenty-five minutes. Then, with a broom made of the leaves and fine twigs of birch, the surface of the body is flagellated in order to stimulate the skin; after which, the patient wipes himself, dresses, and takes a brisk walk.

In the oriental bath not only are these operations practiced, but added to them is a shampooing of all the muscles, after applying soap over the whole surface, and this is followed by washing the skin several times, and, finally, friction with essence.

In the employment of these baths what ought especially to occupy the attention of the physician are the effects on the circulation. These consist, with some persons, of beatings in the head more or less violent; with others, of palpitations, and with others of a sense of oppression. The two former are almost always soothed, first by baths less heated, and second, by the repeated use of the cold sponge applied upon the head and over the region of the heart. As regards the oppression it is only temporary, and it is a matter of observation that the persons who bear with difficulty vapor baths, bear with still greater difficulty the weight of water in bathing houses. However, as the height from which the water falls in the latter can be diminished, which we cannot do as regards the vapor bath, it follows that it is necessary to use much moderation in applying the latter in such cases.

I have never known grave accidents to occur in a vapor bath, except they were the results of imprudence or negligence.

A circumstance important to be kept in mind is, that a vapor bath may be taken after eating. Indeed, it is better that it should not be taken fasting, but after soup or broth. In a word, these baths do not disturb digestion as do water baths.

As respects vapor *douches*, they are given by means of a tube joined to a conducting pipe. They are always given *en jet*. It is customary to take them as warm as possible.

